# Fragmented QRS Electrocardiogram - The Hidden Talisman?

**Published:** 2009-09-01

**Authors:** Frijo Jose, Mangalath Krishnan

**Affiliations:** 1 Senior Resident, Department of Cardiology, Calicut Medical College, Kerala, India; 2 Professor and Head, Department of Cardiology, Calicut Medical College, Kerala, India

**Keywords:** Fragmented QRS

## Introduction

There are several stigmas on the resting surface electrocardiogram that are indicators of past myocardial injury. Broad QRS pattern with bundle branch block, Q waves, persistent ST elevation are some of those facsimiles which may at times even be considered as definitive signs of left ventricular impairment.

We would like to focus here on a lesser known entity of the surface electrocardiogram - the fragmented QRS complex. This marker of myocardial injury may often be the only electrocardiographic marker in patients with non-Q myocardial infarction and in patients with resolved Q wave. It can also be a reliable pointer to left ventricular functional compromise.

Fragmented QRS electrocardiograms were for the first time recorded from canine hearts with experimentally induced acute ischemia and healing. It was found that fragmented electrocardiograms were more frequently observed in healed myocardial infarctions more than 2 weeks old, than in preparations from 5 day old infarcts [1].

The asynchronous excitation of muscle fibers causing fragmentation of electrocardiogram may be due to the poorly inter-connected muscle bundles; separated by high resistance intercellular connective tissue caused by the healing process. This effect is most pronounced at the bordering areas of necrosis where the connective tissue invades the surviving 'islands' of muscle, hence causing separation and distorted orientation of muscle fibers. Any form of gross structural abnormality, like large chamber dilatation, may also cause a similar picture. Flowers et al  [2] have even suggested the high frequency notching of QRS complex as a screening device for any structural heart disease causing biventricular enlargement.

## Ischemic heart disease and fragmented QRS

It has been observed in various studies  [3,4] that fragmented QRS on the resting electrocardiogram has a moderate sensitivity (62.2%) and high specificity (up to 94%) in detecting ischemic heart disease. It is especially relevant in cases where the baseline electrocardiogram does not have a Q wave. The presence of a Q wave in addition to QRS fragmentation further augments the sensitivity of detecting ischemic heart disease up to 92.4%. Many studies have reiterated the significance of the fragmented QRS on patients with resolved Q waves and non-Q myocardial infarction.

## Myocardial scar, left ventricular aneurysm and fragmented QRS

Das MK et al  [5] using myocardial perfusion imaging have shown that fragmented QRS has a superior sensitivity and negative predictive value compared to Q waves in detecting myocardial scar; though there was a small compromise in specificity - especially in inferior wall myocardial necrosis. The presence of rsR' pattern or its variants on the left sided precordial leads was found to be an excellent marker of extensive confluent scarring and hence, ventricular aneurysm  [6]. The sensitivity and specificity of f-WQRS in detecting myocardial scar is 86.8% and 92.5% respectively  [7].

Broad premature ventricular complexes (≥160 ms) with notched QRS (notch separation >40 ms) was found to be a reliable marker of a global form of ventricular dysfunction involving ventricular mass, chamber size or function   [8]. It may also be indicative of the chronic nature of the underlying disease.

## Localization value of fragmented QRS

Flowers et al   [9] found a certain amount of localizing value of fragmented QRS, especially in patients without chamber dilatation in the absence of Q waves. This can be useful in patients with resolved Q waves and non transmural myocardial infarction. Postero-inferior lesions are more reliably localized (inferior axis leads) than anterior lesions (lesions are larger and the periphery may extent laterally).

## Left ventricular function and fragmented QRS

Fragmented QRS electrocardiogram is an independent predictor of left ventricular function. It is a marker of higher stress myocardial perfusion abnormalities and functional deterioration   [5]. This was also observed in other studies, where gross left ventricular dilation and decreased ejection fraction were found to be faithfully reflected by the fragmented electrocardiogram   [2,6].

## Fragmented QRS and prognosis

QRS fragmentation with or without Q waves was found to predict a higher mortality and recurrent cardiac events than either Q wave  alone or resolved Q wave without QRS fragmentation   [10,11]. Hence fragmented QRS, though not extensively studied yet, is probably a reliable indicator of past myocardial ischemia in the absence of Q waves. It also suggests increased scar burden and poorer prognosis. This promising and simple noninvasive modality of investigation may be of immense help in evaluating coronary artery disease patients, but needs to be energetically promoted in routine clinical practice, where it is a neglected entity at present.

## Conclusion

Fragmented QRS on the resting surface electrocardiogram is a simple, fast and inexpensive modality of non invasive investigation that can be of great value in predicting the cardiac status and prognosis of an individual being evaluated for coronary artery disease.
